# Dietary Mannan Oligosaccharides Modulate Gut Inflammatory Response and Improve Duodenal Villi Height in Post-Weaning Piglets Improving Feed Efficiency

**DOI:** 10.3390/ani10081283

**Published:** 2020-07-28

**Authors:** Alessandro Agazzi, Vera Perricone, Fabio Omodei Zorini, Silvia Sandrini, Elena Mariani, Xian-Ren Jiang, Alessandra Ferrari, Maurizio Crestani, Thi Xuan Nguyen, Valentino Bontempo, Cinzia Domeneghini, Giovanni Savoini

**Affiliations:** 1 Department of Health, Animal Science and Food Safety “Carlo Cantoni” (VESPA), Università degli Studi di Milano, Via dell’Università 6, 26900 Lodi, Italy; alessandro.agazzi@unimi.it (A.A.); fabio.omodei@unimi.it (F.O.Z.); silvia.sandrini@unimi.it (S.S.); thixuan.nguyen@unimi.it (T.X.N.); valentino.bontempo@unimi.it (V.B.); cinzia.domeneghini@unimi.it (C.D.); giovanni.savoini@unimi.it (G.S.); 2 Dipartimento di Scienze Medico-Veterinaire, Univeristy of Parma, Via del Taglio 10, 43126 Parma, Italy; elena.mariani@unipr.it; 3 Feed Research Institute, Chinese Academy of Agricultural Sciences, Zhongguancun South Street 12, Beijing 100081, China; jiangxianren@caas.cn; 4 Department of Pharmacological and Biomolecular Sciences, Università degli Studi di Milano, Via Balzaretti 9, 20133 Milano, Italy; aferrari@g.ucla.edu (A.F.); maurizio.crestani@unimi.it (M.C.)

**Keywords:** mannan oligosaccharides, post-weaning piglets, gut health, gene expression, intestinal morphology, faecal microbiota

## Abstract

**Simple Summary:**

Postweaning is a stressful period for piglets, accompanied by several modifications of the gastrointestinal tract, which can impair both animal health and performance. Nowadays, some classes of feed additives are under evaluation to benefit health status and promote growth in farm animals, modulating the development of the gastrointestinal tract and the residing microflora, and ameliorating the immune response during stressful situations. In the present study, we investigated the efficacy of mannan oligosaccharides (MOS) to support gut health and improve growth performance. Our results suggest that MOS can exert beneficial effects on gut health, improving duodenal morphology and modulating the expression of inflammation-related genes, which are accompanied by improved feed efficiency.

**Abstract:**

The aim of this study was to evaluate the effects of mannan oligosaccharides (MOS) on gut health and performance in post-weaning piglets. In total, 40 piglets were divided into two experimental groups and fed a basal diet with (TRT) or without (CON) 0.2% mannan oligosaccharides for 35 days. Growth performance was determined weekly and faecal microbial composition on days 0, 14 and 35. On day 36, histometrical evaluations were performed on duodenal, jejunal, ileal, and colon samples. mRNA gene expression of inflammation-related genes was evaluated in samples of ileal Peyer’s patches (IPP). MOS administration improved feed efficiency in the last two weeks of the trial (*p* < 0.05), and a decreased clostridia content was found in faeces at day 14 (*p* = 0.05). TRT piglets showed increased duodenal villi height (*p* < 0.05), and reduced mRNA levels of Tumour Necrosis Factor α (*p* < 0.05) and Toll-Like Receptor 4 (*p* < 0.01) in IPP. Our results suggest beneficial effects of MOS supplementation on gut morphology and the expression of inflammation-related genes in post-weaning piglets, accompanied by increased feed efficiency.

## 1. Introduction

Optimal conditions of the gastrointestinal tract (GIT) throughout all the production cycle are of primary importance for the piglet metabolism and physiology to improve performance [1]. In this light, the interest toward ‘gut health’ in animals has gained significant attention over the last decade. It is generally accepted that the concept of ‘gut health’ encompasses several physiological and functional features, including effective digestion and absorption of nutrients, proper structure and function of the gut barrier, balanced and stable microbial populations, and effective function of the intestinal immune system [2]. Gut health has been widely investigated in relation to the post-weaning period, which is a stressful moment in pig’s life, frequently accompanied by several modifications of the GIT. In this phase, morphological changes [3], high epithelial cell turnover rates [4] and modifications of the gut microbiota mass and composition [5] are often associated with an impairment of the digestive, absorptive, and immune functions of the GIT [1], leading to low performance.

Antimicrobial molecules have been largely employed in the past to overcome gut health impairment due to weaning, and therefore to promote animal performance. However, the increasing awareness on the antimicrobial resistance associated risks led to the ban of their inclusion in the feed for auxinic purpose; this was then translated in moving research community’s efforts toward the investigation of potential alternatives [6]. With the purpose to sustain animals’ health status and performance, the supplementation of different molecules, compounds and bioactive substances have been investigated in recent years. Nowadays, several classes of feed additives are available and widely employed in pig farming such as probiotics [7], prebiotics [8], postbiotics [9], nucleotides [10] and phytobiotics [11].

Among the potential alternatives to antimicrobial growth promoters, mannan oligosaccharides (MOS), that are included in the EU feed material register, have received particular attention. MOS are a glucomannan complex derived from the yeast cell walls that cannot be hydrolized by digestive enzymes of the GIT. Despite formally belonging to the class of prebiotics, i.e., non-digestible food ingredients that promote the growth of beneficial microorganisms in the intestine [12], the mode of action of MOS slightly differs from the other class compounds [13]. MOS can modify the microbiota composition through an indirect mechanism, rather than acting as a direct nutrient for the intestinal microbial populations. Specifically, MOS are able to prevent the adhesion of pathogenic bacteria to intestinal epithelial cells by attachment to the mannose-binding proteins expressed on the bacterial fimbriae [14]. Via the modulation of gut microbiota, MOS can also improve some morpho-functional aspects of the GIT [15,16,17]. The administration of MOS has been associated with the proliferation of beneficial microbes, including lactic acid bacteria [18,19]. Lactobacilli in turn can indirectly stimulate growth and development of the enterocytes, exerting a trophic action [20].

Beyond their ability to modulate gut microbiota, MOS supplementation evidenced additional beneficial properties such as decreased incidence of diarrhoea [21,22] and higher growth performance [23,24]. Usually, the most significant results on growth when supplementing MOS in swine are obtained during the first and second week just after weaning [25]. Observed improved performance or feed efficiency seem to be related to the improvement of disease resistance against pathogenic microbes, enabling a low immune status [26] and maximizing nutrients utilization for growth, rather than for the activation of the immune system [27].

A large body of literature has also evidenced the efficacy of dietary MOS to enhance pigs’ immunity by the increase in the immunoglobulin serum concentrations [28], the stimulation of the mucosal immunity [21], and the modulation of the inflammatory response [29]. Duan et al. [29] reported decreased serum concentration of proinflammatory cytokine and increased circulating levels of anti-inflammatory cytokine Interleukin-10 in young piglets following the administration of 0.8 mg/kg BW of MOS. However, at present there is still a lack of knowledge about the modulation of inflammation-related gene expression in the GIT mucosa of postweaning piglets when supplemented with MOS.

The present study was designed to evaluate the efficacy of dietary MOS supplementation to improve gut health and performance. To assess the effects of the dietary treatment on gut health, we evaluated the gene expression of some Interleukins (ILs) and Toll-Like Receptors (TLRs) in addition to intestinal morpho-functional parameters and faecal microbial populations.

## 2. Materials and Methods 

### 2.1. Experimental Design, Animals, and Housing

The trial was performed at the Animal Production Research and Teaching Centre of the Polo Veterinario, Università degli Studi di Milano (Lodi, Italy). All the experimental procedures were reviewed and approved by the Animal Care and Use Committee of the University of Milan (Protocol No. 15/12).

A total of 40 crossbred (Stambo HBI × Dalland) early-weaned male piglets (24 days of age), with similar body weight (BW) (6.78kg ± 0.33 kg), were obtained from a commercial swine herd and enrolled in a 35-days experiment. At day 11 and 19 post-partum, piglets were vaccinated against *Mycoplasma hyopneumoniae* (Ingelvac Mycoflex, Boehringer Ingelheim, Ingelheim, Germany) and *Porcine circovirus type 2* (Ingelvac Circoflex, Boehringer Ingelheim, Ingelheim, Germany).

At the arrival at the Research Centre, all animals were housed in one room equipped with computer-controlled heating and mechanical ventilation systems. The room temperature was maintained at 28 °C during the first week, with ventilation rate of 10 m^3^/h/head. The temperature was gradually reduced 1 °C per week, to a final temperature of 24 °C at the end of the trial. Piglets were reared in a total number of 20 pens equipped with plastic slatted floor, a one-sided self-feeder and a nipple-watering device to provide the piglets with ad libitum access to feed and water throughout the experimental period.

The animals were randomly allotted to two experimental groups, homogenous for initial BW. Each group was composed of 10 replicates (pens), with two piglets allocated to each pen. The experimental groups consisted of a control group (CON), receiving the basal diet, and a treated group (TRT), fed the basal diet with the inclusion of 0.2% MOS (AgriMOS^®^, Lallemand SAS, Blagnac Cedex, Toulouse, France). Both CON and TRT diets did not contain antimicrobials or growth promoters and were designed to be isocaloric and isoproteic. The basal diet was formulated to meet or exceed the nutrient requirements of weaned piglets according to the National Research Council requirements [30] (Table 1), and was provided in meal form by Tracciaverde s.r.l. (Bonemerse, Cremona, Italy). MOS were included in the diet through the substitution of an equivalent amount of wheat meal. The chemical composition of the diets was analysed at the beginning of the trial. Moisture, crude protein, ether extract, crude fiber, and ash were determined following the Association of Official Analytical Chemists methods of analysis (AOAC, 2005). Lysine and methionine content were calculated by AMINODat 4.0 (Evonik Nutrition & Care GmbH, Essen, Germany). Calcium, total phosphorus and metabolizable energy contents in the diet were calculated by INRA-CIRAD-AFZ feed tables. Available online: https://feedtables.com/content/tables (accessed on 30 June 2020).

### 2.2. Growth Performance and Faecal Microbiological Assay

Individual BW was recorded weekly from d 0 of the trial using an electronic scale (Ohaus ES100L, Pine Brook, NJ, USA; sensitivity ± 0.02 kg) and feed intake (FI) was recorded daily for each pen. Subsequently, weekly body weight gain (BWG) and feed efficiency (gain:feed ratio, G:F) were calculated. Faecal score was recorded daily on pen basis for the first 14 days of the trial, and then weekly (days 21, 28 and 35) until the end of the experimental period. Higher frequency of evaluation was employed during the first two weeks due to the high sensitivity of the animals to develop gastrointestinal disorders in this phase. Faecal score was evaluated by means of a subjective 5-point faecal consistency scoring system: 1 = hard, dry pellet; 2 = firm, formed stool; 3 = soft, moist stool that retains shape; 4 = soft, unformed stool; 5 = watery liquid that can be poured. Liquid consistency (score 4 to 5) was considered indicative of diarrhoea [31], while a score equal to 3 represented the ideal faecal consistency. On days 0, 14, and 35 faecal samples (10 g) for microbiological assay were collected from the same one piglet per pen, randomly selected at the beginning of the trial. The samples were collected in the morning (0900 h), before the procedure of animal weighing started. All samples were collected in aseptic conditions, stored at +4 °C, and transferred to the laboratory for the analysis. Faecal clostridia, lactobacilli, and coliforms contents were determined. Each faecal sample was diluted with 90 mL of sterile saline; then, 10-fold dilutions using sterile saline were prepared and plated onto the different media. Clostridial faecal contents were enumerated onto plate count Tryptose Sulphite Cycloserine Agar (TSC, Oxoid, Basingstoke, UK). The media was incubated in jars at 37 °C for 48 h using anaerobiosis generators (AnaeroGen, Oxoid, Basingstoke, UK). The faecal content of lactobacilli was determined by De Man, Rogosa, Sharpe agar (MRS agar) that was incubated at 37 °C for 72 h in jars in microaerophilic conditions (10% CO_2_). Finally, the faecal coliform content was determined using Violet Red Bile Agar (VRBA, Oxoid, Basingstoke, UK) incubated at 44 °C for 24 h. The lactobacilli/coliforms ratio was calculated as the log difference of lactobacilli and total coliform contents [32].

### 2.3. Intestinal Histometry

At d 36 of the trial, one piglet per pen was selected and sacrificed. Within each pen, the selected animal was the one with BW closer to the mean of the relative experimental group. After 16 h of starvation, animals were electrically stunned and bled, and samples were collected. Intestinal tissue samples were collected from all sacrificed animals for histometric evaluations. Samples were collected from the proximal duodenum (7 cm below the pylorus), the middle jejunum (the middle area was established by counting the jejune folds and sampling the fold at the midpoint), the distal ileum (5 cm above the ileocecal valve), and the proximal colon (first fold of the ascending colon). Collected samples were fixed by immersion in 4% paraformaldehyde in 0.01 M phosphate-buffered saline (pH 7.4) for 24 h at 4 °C, dehydrated in a graded series of ethanol, cleared with xylene and embedded in paraffin. A microtome section (4 µm thick) was obtained from each sample, processed in low-melt paraffin and, after being deparaffinised and rehydrated, stained with haematoxylin and eosin and examined under light microscope. The following parameters were evaluated for 10 villi or 10 crypts for each stained section: villus height, villus width, number of goblet cells per villus, crypt depth, crypt width, and number of goblet cells per crypt. The length of the villi was measured from their base (transition zone between villus and crypt) to their apex, and the depth of the crypts was measured from the base of the villi to the bottom of the glands. Measurements were taken at 200X using an Olympus BX51 photomicroscope equipped with the DP software for image analysis (Olympus, Italy). Subsequently, villus height to crypt depth ratio was calculated, and the thickness of the tunica propria for 10 randomly selected intestinal areas at 200X per section was determined. In order to eliminate any experimental bias, the histometrical examinations were blinded.

### 2.4. ILs and TLRs Gene Expression in IPP

Ileal segments containing Peyer’s patches were obtained approximately 5 cm before the ileocecal valve from each sacrificed piglet. Tissues were cut longitudinally along the side of the intestine opposite the Peyer’s patches, gently rinsed with saline solution and stripped of the underlying smooth muscle layer. Peyer’s patches were then excised from the tissue samples with a lancet. Tissue samples of approximately 10 mg were collected from the isolated ileal Peyer’s patches (IPP), immediately stored in 1.5-mL cryovials with 0.9 mL RNAlater solution (Invitrogen, Life Technologies Ltd., Paisley, UK) and frozen at −20 °C. The levels of IL-1α, IL-1β, IL-6, IL-10, TNF-α, TLR2, TLR4, and the reference genes β-actin and glyceraldehyde 3-phosphate dehydrogenase (GAPDH) in the samples of IPP were subsequently measured. The total RNA was double extracted with TRIzol Reagent (Invitrogen, Life Technologies Ltd., Paisley, UK), purified with a commercial kit (Macherey-Nagel, Oensingen, Switzerland) and quantified using a NanoDrop spectrophotometer (Thermo Scientific, Waltham, MA, USA). Specification for RT-qPCR thermal protocol, primers, and probes for gene expression is given in Appendix A.

The relative expression levels of the target genes were assessed using a standard four-point (1000, 200, 40, and 8) five-fold-diluted curve. The standard curve was generated with increasing amounts of cDNA. The quantity values of the target genes were normalized to the quantity values of the reference genes β-actin and GAPDH of *S. scrofa*. The levels of β-actin and GAPDH mRNA were comparable in all samples of all the animals. All the samples have been run in triplicates.

### 2.5. Statistical Analysis

In the present trial, a completely randomized design was used. Growth performance, faecal score, faecal microbiology, and mRNA gene expression were analysed using one-way analysis of variance (ANOVA) to compare the means of the two groups applying a General Linear Model (GLM) procedure of Statistical Analysis System Software (SAS version 9.4, SAS Institute Inc., Cary, NC, USA). The model included the treatment as a fixed effect and the pen or the piglet as a random effect. The pen represented the experimental unit for growth performance, while the piglet represented the experimental unit for the remaining assays.

Histometric data on villus height, crypt depth, villus height to crypt depth ratio, villus width, crypt width and tunica propria thickness were analysed using a MIXED procedure (SAS Inst. Inc., Cary, NC, 2006) considering the piglets as the experimental unit. Because of the significant differences in villus height, the goblet cell counts were analysed by covariance to refine the analysis of the overall treatment difference. All numerical data in tables are presented as least-square means (LSMeans) accompanied by standard error of the mean (SEM) values. Differences between groups were considered statistically significant at *p* ≤ 0.05.

## 3. Results

### 3.1. Growth Performance and Faecal Microbiology

Mannan oligosaccharides supplementation did not affect BW, BWG and FI, while feed efficiency was improved in TRT piglets in the last two weeks of the trial (*p* = 0.03 and 0.04, respectively) (Table 2).

No incidence of diarrhoea was observed in the experimental groups. MOS fed piglets evidenced a better faecal consistency at the beginning (3.1 vs. 3.3 ± 0.12; *p* = 0.03) and on day 5 (3.1 vs. 3.4 ± 0.11; *p* = 0.04), while at the end of the trial TRT animals had firmer faeces than CON (2.1 vs. 2.5 ± 0.11; *p* = 0.04).

Faecal bacterial counts showed TRT had lower lactobacilli content and lactobacilli/coliforms ratio at the beginning of the trial (*p* < 0.01 and *p* = 0.02; respectively), while a decreased faecal clostridia content in MOS fed piglets was detected on day 14 (*p* = 0.05) (Table 3). No significant variations on faecal coliforms were observed during the trial among the experimental groups.

### 3.2. Intestinal Histometry, ILs and TLRs Gene Expression in IPP

**Figure 1:**
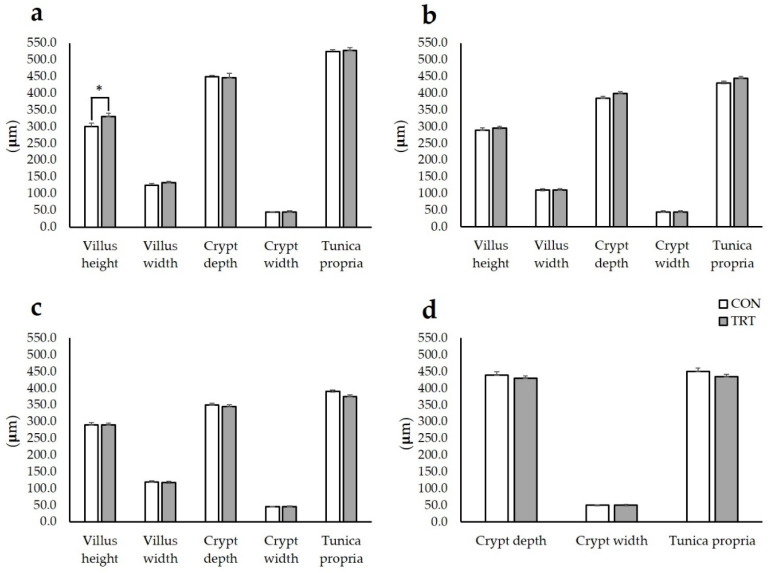
Effects of mannan oligosaccharides supplementation in post weaning piglets on GIT histometry. Data shown as LSMeans ± SEM. (**a**) duodenum, (**b**) jejunum, (**c**) ileum, and (**d**) colon; CON (n = 10): animals receiving the basal diet with no MOS supplementation; TRT (n = 10): animals receiving the basal diet with 0.2% MOS supplementation; LSMeans: least-square means; SEM: standard error of the mean; * significant at *p* < 0.05.

MOS supplementation led to a significant increase in duodenal villus height (328.78 μm vs. 296 μm ± 9.0 μm; *p* < 0.01), while villus width, crypt depth and width, as well as tunica propria were not affected by MOS administration in all the considered intestinal segments (Figure 1). Similarly, villus height to crypt depth ratio and the number of goblet cells/villi adjusted for villus height were not significantly different between the two experimental groups (Table S1).

**Figure 2:**
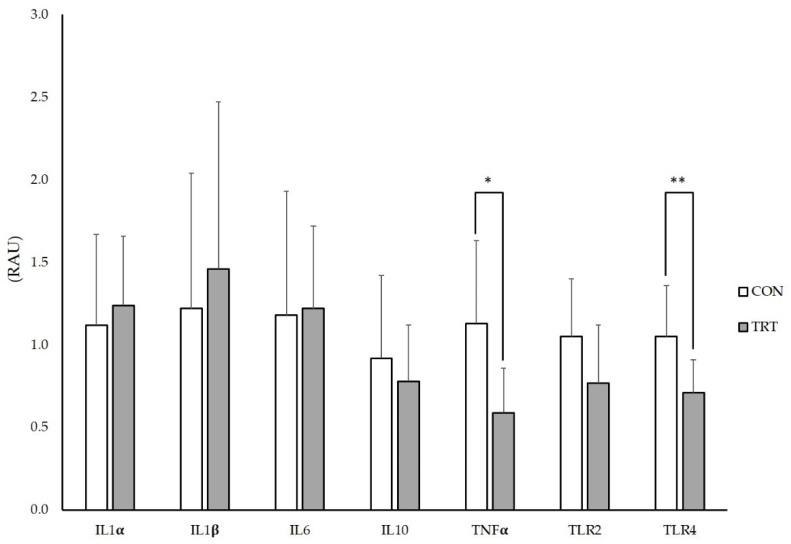
mRNA expression levels of genes encoding intestinal pro-inflammatory mediators in ileal Peyer’s patches of postweaning piglets supplemented with mannan oligosaccharides. Data shown as LSMeans ± SEM. Values are expressed as relative arbitrary unit (RAU). CON (n = 10): animals receiving the basal diet with no MOS supplementation; TRT (n = 10): animals receiving the basal diet with 0.2% MOS supplementation; PCR reactions were normalised to the Ct values of the reference genes β-actin and glyceraldehyde 3-phosphate dehydrogenase (GAPDH) of *S. scrofa*, and differential expressions were determined using the comparative Ct method; LSMeans: least-square means; SEM: standard error of the mean; * significant at *p* < 0.05; ** significant at *p* < 0.01.

Quantification of mRNA performed on IPP revealed a significant reduction in TNF-α (*p* < 0.05) and TLR4 (*p* < 0.01) in the TRT group, compared to the CON group. No differences were found for IL-1α, IL-1β, IL-6, IL-10 and TLR2 mRNA expression levels between the groups (Figure 2).

## 4. Discussion

In the present study, the hypothesis that MOS dietary supplementation can improve the gut health and performance of postweaning piglets was tested. Our results showed a higher duodenal villi height and reduced TNFα and TLR4 expression levels in the ileum Peyer’s patches. These results were mirrored by increased feed efficiency during the last two weeks of the trial.

The sustainment of the eubiosis of the GIT via the reduction in pathogen colonization and the promotion of the growth of beneficial bacteria such as Lactobacilli [13] has been proposed as the main mechanism of action of MOS. Their efficacy seems, however, to be variable [16,19,29,33] as highlighted by our results, where MOS administration evidenced only minor effects on faecal bacterial population. At the beginning of the trial, lactobacilli faecal content was significantly lower in the TRT group, and consequently the lactobacilli/coliforms ratio was also. This evidence is clearly not related to the experimental treatments, but could reflect differences in the maternal microbiome and milk composition [34,35], that unfortunately were not a selection criteria of the animals involved in the present study. Even though no significant differences in lactobacilli faecal counts were pointed out between the two experimental groups on days 14, it is worth nothing that the lactobacilli count increased in TRT in the first two weeks of the trial contrary to CON. Such a recovery of lactobacilli could suggest a prebiotic-like effect of MOS, but contrasting results regarding the modulatory activity of MOS over lactic acid bacteria are reported in literature. Castillo et al. [16] observed a higher lactobacilli:enterobacteria ratio in the jejunum content of weaning piglets after 14 days administration of 0.2% MOS, but only when MOS were administered in association with Zn. On the contrary, the administration of yeast as a source of MOS increased the faecal lactobacilli concentration of post-weaning piglets after 28 days administration, but no differences were observed at day 14 [19].

The lack of a strong effect of MOS over the faecal bacterial population may be due to the high hygienic experimental conditions applied in our experimental facilities. The ability of MOS to improve microbial ecology and reduce the number of pathogenic bacteria in fact seems to be exacerbated when an exposure to a pathogen is provided, as in challenging conditions [13].

MOS supplementation has been first reported to improve some morpho-functional aspects of the intestine in chickens, with consequent health benefits [36,37]. At present, a lower number of studies has been conducted in piglets, reporting similar beneficial effects [13,15,38] or no effects on gut morphology [33,39]. In the present trial, MOS administration increased duodenal villi height at day 36, but no other differences were observed. These results corroborate with the minor changes observed in faecal microbial population. The beneficial effects of MOS on GIT morphology have been primarily attributed to the modulation of the residing microflora, reducing the pathogen load and its harmful consequences for the enterocytes, and/or stimulating the proliferation of beneficial bacteria [13]. As previously stated, the minor effects of MOS administration could also suggest high hygienic conditions of the experimental facilities and consequent low pathogenic load [33]. This hypothesis was further corroborated by the lack of difference in the goblet cells, whose increase is usually indicative of a defence reaction of the intestine toward a pathogen exposure [40]. Therefore, our results suggest the absence of a pathogenic arousal.

The postweaning period is recognized as one of the most stressful stages in a pig’s life [3]. This phase is often associated with an inflammatory status of the intestinal mucosa that can compromise villous crypt structure [1] and may contribute to both anatomical and functional intestinal disorders [41]. A persistent inflammatory response, accompanied by excessive production of pro-inflammatory cytokines is responsible for the activation of a cascade of events that can lead to a reduction in animals performance [42]. Besides the role played by the exposure to new dietary antigens, the activation of the inflammatory response at weaning can be referred to a state of dysbiosis, commonly accompanying the weaning transition [43]. Such a response is mediated by the activation of TLRs, whose engagement leads to the activation of several signalling pathways, finally resulting in the production of proinflammatory cytokines [44]. Among others, TLR2 and TLR4, which are highly expressed in IPP [45], are of main importance as they are able to recognize a diversified array of pathogenic ligands [46].

MOS have been proposed to improve gut morphology through a secondary and indirect mechanism of action consisting in the modulation of the inflammatory response [37], thus supporting animal health and performance. In the present study, the administration of MOS during the postweaning phase modulated the expression of inflammation-related genes in the IPP of the intestinal mucosa, such as TNF-α and TLR4, thus partially supporting a reduced stimulation of the inflammatory response. Ileum is highly susceptible to pathogens and its structural and functional changes linked to nutraceutical administration are of a predictive value to evaluate intestinal defensive responsiveness [47]. Published papers targeted the ileum rather than jejunum when evaluating the efficacy of feed additives with presumed beneficial effect on performance and inflammatory- and immune-related parameters [10,48] because in differentiated and matured ileum, immunological structures like Peyer’s patches are much more abundant [49]. The significant reduction in TLR4 expression in IPP we observed in our trial could be explained by a reduced exposure of the enterocytes to pathogenic bacteria thanks to the decoy role of MOS. Mannan oligosaccharides can indeed prevent the adhesion of type 1 fimbriae bacteria to the enterocytes by binding itself to the bacteria [14]. This mechanism of action supports the observed reduction in TLR4 expression, contrary to the lack of differences on TLR2. Type-1 fimbriae are mannose-specific lectin expressed by many Gram-negative bacteria [50], and TLR4 is mainly involved in the recognition of this class of bacteria, while TLR2 is involved in the recognition of Gram-positive bacteria [46]. Moreover, TLR-4 is able to modulate the expression of pro-inflammatory cytokines and related genes such as TNF-α, IL-1, and IL-6 [51]. In the present trial we observed only a reduction for TNF-α, while no differences were found in the expression of IL-1α, IL-1β, IL-6 and IL-10. To the best of our knowledge, only Duan et al. [29] have investigated the role of MOS in the modulation of gene expression of inflammation-related genes, but in the intestinal mucosa of the jejunum, showing no effect on the mRNA expression of TLR2, TLR4, IL-8, NF-κB p65 and PPAR-γ. Beside the gene expression of intestinal immune-related genes, some other studies considered the effects of MOS supplementation in both neonatal and weaned piglets reporting reduced serum concentration of TNF-α [52], IL-2, IL-4, INF-γ, and increased levels of IL-10 [29]. In our trial, interleukins gene expression was not affected by MOS dietary administration; this could be partially explained by the absence of an immune challenge as previously described, which usually appears to be a significant factor affecting these outcomes [13].

Given the role of MOS in the modulation of several aspects of the gut health, improved performance was expected in the present trial. Literature reports as significant improved performance in most of the cases happens during the first fifteen days after weaning when supplementing MOS in swine [23,25]. In our trial, we observed an increased feed efficiency in TRT only in the last two weeks of the trial, but results between different trials seem anyway quite variable depending on different factors [23]. Previous works [26] suggest as the higher G:F ratio in TRT in our trial could be related to MOS’s ability to improve disease resistance against pathogenic microbes, promote beneficial bacteria in the GIT, and to enable a low immune status. This was partially evidenced in the present study through the recovery of faecal Lactobacilli content and the decrease in inflammation-related gene expression in IPP. Moreover, the positive results evidenced in the histometric assays could have participate to a higher ability in the absorption of nutrients. Taken together, all these aspects could have led to a higher nutrients utilization for growth, rather than for the activation of the immune system [27]. This result represents an interesting finding from a commercial perspective since feed efficiency strongly impacts farm profitability as feed is the major cost in pig rearing production [53]. However, in the present trial, the increased feed efficiency was not sufficient to be turned into higher gain or final BW in TRT piglets, partially in contrast with some published works [23,28]. Different variables can be accounted for the minor results on the overall growth performance. It is generally recognized that the efficacy of feed additives such as prebiotics and probiotics is increased when environmental or immunological challenges are performed [19], and the age of the animals at weaning could contribute as well. Che et al. [52] reported increased G:F in piglets receiving dietary MOS only after the inoculation of the animals with Porcine Reproductive and Respiratory Syndrome Virus (PRRSV), while Miguel et al. [23] showed that the feed intake response to MOS administration was larger for piglets weaned at an earlier age (17 to 18 days) than at a later stage (24 to 28 days), as in our case.

## 5. Conclusions

Our results suggest beneficial effects of MOS supplementation in postweaning piglets, increasing duodenal villi height and reducing the expression of inflammation-related genes at IPP level. In accordance with an improved gut health condition, positive effects were also observed on production traits, with increased feed efficiency toward the end of the experimental period. However, the optimal experimental conditions might have masked the effects of MOS; therefore, additional studies including more challenging conditions will help to clarify the real potential of MOS administration to post-weaning piglets.

## Figures and Tables

**Table 1:** Feed ingredients and nutrient composition of the post-weaning experimental diets.

Ingredients (% as Fed)	Dietary Treatment	
	CON	TRT
Wheat meal	33.80	33.60
Corn meal	24.00	24.00
Soybean meal, 44% CP	22.10	22.10
Wheat bran	10.00	10.00
Soy protein concentrate ^1^	3.00	3.00
Soy oil	3.00	3.00
Dicalcium phosphate, 18%	1.00	1.00
Animal fat	1.00	1.00
Calcium carbonate, 37%	0.80	0.80
L-Lysine HCl, 78%	0.30	0.30
Sodium chloride	0.30	0.30
Vitamin premix ^2^	0.30	0.30
Mannan oligosaccharides ^3^	-	0.20
L-Threonine, 98%	0.15	0.15
DL-Methionine, 98%	0.10	0.10
Sweetener ^4^	0.05	0.05
Triptophane, 98%	0.05	0.05
Flavour ^5^	0.05	0.05
Chemical composition ^6,7^ (% DM)		
Moisture	11.50	11.42
Crude protein	22.33	22.11
Ether Extract	4.82	4.90
Crude Fiber	3.15	2.97
Ash	6.10	7.01
Ca	0.76	0.75
Total P	0.71	0.70
Lysine	1.42	1.44
Methionine	0.51	0.50
ME (Mcal/kg)	3.82	3.78

^1^ The soy protein concentrate contained 650 g/kg of CP, 65 g/kg of Lys and 70 g/kg of crude fat. ^2^ Provided per kg of complete diet: vitamin A, 10 000 IU; vitamin D3, 1 000 IU; vitamin E, 50 mg; vitamin B1 1.0 mg; vitamin B2 3.0 mg; vitamin B6 3.0 mg; vitamin B12, 0.03 mg; riboflavin, 9 mg; pantothenic acid, 14 mg; nicotinic acid, 15 mg; biotin, 0.06 mg; vitamin PP, 0.35 mg; folic acid, 0.97 mg; vitamin K3, 3 mg; choline, 300 mg; Fe, 100 mg; Cu, 20 mg; Co, 0.75 mg; Zn, 100 mg; Mn, 10 mg; I, 0.85 mg; Se, 0.4 mg; ethoxyquin, 150 mg. ^3^ AgriMOS^®^, Lallemand SAS, Blagnac, France. ^4^ Dextrose monohydrate, Rouquette, France. ^5^ Luctarom SFS, Feedland Group, Moscow, Russia. ^6^ Calcium, total phosphorus and metabolizable energy (ME) contents in the diet were calculated by INRA-CIRAD-AFZ feed tables. ^7^ Lysine and methionine content were calculated by AMINODat 4.0 (Evonik Nutrition & Care GmbH, Essen, Germany).

**Table 2:** Effects of mannan oligosaccharides supplementation on growth performance of postweaning piglets. Data are shown per pen as LSMeans ± SEM.

Item	Days of Treatment	Dietary Treatment			*p*-Value
		CON	TRT	SEM	
BW (kg)	0	13.58	13.54	0.65	0.96
	7	13.84	14.05	0.66	0.83
	14	16.68	16.72	0.87	0.98
	21	22.06	21.66	1.12	0.80
	28	27.07	27.4	1.18	0.85
	35	33.73	34.43	1.51	0.75
BWG (kg)	0–7	0.26	0.51	0.21	0.41
	7–14	2.84	2.67	0.29	0.68
	14–21	5.38	4.94	0.36	0.40
	21–28	5.01	5.74	0.29	0.09
	28–35	6.66	7.03	0.48	0.60
	0–35	20.15	20.89	1.12	0.65
FI (kg)	0–7	2.84	2.86	0.21	0.94
	7–14	4.99	5.21	0.08	0.07
	14–21	7.61	7.34	0.27	0.48
	21–28	9.35	8.93	0.39	0.46
	28–35	13.51	12.28	0.47	0.08
	0–35	38.30	36.63	1.17	0.32
G:F	0–7	0.06	0.14	0.08	0.47
	7–14	0.57	0.51	0.05	0.43
	14–21	0.70	0.67	0.03	0.55
	21–28	0.54	0.65	0.03	0.03
	28–35	0.49	0.57	0.025	0.04
	0–35	0.52	0.57	0.02	0.08

Note: BW: body weight; BWG: weekly body weight gain; FI: weekly feed intake; G:F: gain to feed ratio; CON (n = 10 pens): animals receiving the basal diet with no MOS supplementation; TRT (n = 10 pens): animals receiving the basal diet with 0.2% MOS supplementation; LSMeans: least-square means; SEM: standard error of the mean.

**Table 3:** Faecal bacterial counts of postweaning piglets supplemented with mannan oligosaccharides. Data shown as LSMeans ± SEM.

Bacterial Classes Isolated (log CFU/g)	Days of Treatment	Dietary Treatment			
		CON	TRT	SEM	*p*-Value
Clostridia	0	5.61	5.82	0.30	0.63
	14	3.79	3.00	0.26	0.05
	35	3.24	3.00	0.17	0.33
Lactobacilli	0	9.47	8.52	0.23	<0.01
	14	9.51	10.02	0.18	0.06
	35	9.00	9.41	0.16	0.08
Coliforms	0	8.05	8.48	0.37	0.42
	14	7.16	7.54	0.39	0.50
	35	6.45	6.85	0.29	0.34
Lactobacilli/Coliforms	0	1.43	0.04	0.53	0.02
	14	2.35	2.48	0.63	0.85
	35	2.55	2.57	0.44	0.96

Note: CON (n = 10): animals receiving the basal diet with no MOS supplementation; TRT (n = 10): animals receiving the basal diet with 0.2% MOS supplementation; LSMeans: least-square means; SEM: standard error of the mean.

## Supplementary Materials

The following are available online at https://www.mdpi.com/2076-2615/10/8/1283/s1, Table S1: Effects of mannan oligosaccharides supplementation in post weaning piglets on GIT villus height to crypt depth ratio and goblet cells. Data shown as LSMeans ± SEM.

## Appendix A Gene Expression Analyses

Specific mRNAs were amplified and quantified using the iScriptTM One Step RT-PCR for Probes reagent (Bio-Rad, CA, USA) according to the manufacturer’s instructions. The RT-qPCR analysis was performed with a CFX384 Real-Time System (Bio-Rad, CA, USA). The thermal protocol was as follows: 50 °C for 10 min for reverse transcription and 95 °C for 10 s/60 °C for 30 s for 40 cycles. Proper amplification was checked by performing a melting curve for each PCR product at the end of the last cycle of amplification.

The mixes containing primers and probes for the RT-qPCR are proprietary, and their sequences are not available. The mixes were purchased from Applied Biosystems (Carlsbad, CA, USA) (IL-1α, assay ID: Ss03391335_m1; IL-1β, assay ID: Ss03393804_m1; TNFα, assay ID: Ss03391318_g1; TLR2, assay ID: Ss03381278_u1; TLR4, assay ID: Ss03389780_m1; GAPDH, assay ID: Ss03375435_u1). The set of primers and the probe used for the β-actin quantification (accession number XM_003357928, forward primer 5′-ACTCGATCATGAAGTGCGAC-3′, reverse primer 5′-GTGATCTCCTTCTGCATCCTG-3′, Taqman probe 5′-CGTGTTGGCGTAGAGGTCCTTCC-3′) was designed with IDT software (available online), optimised to work in a one-step protocol and synthesised by Eurofin MWG Operon (Huntsville, AL, USA).
