# Supplementary material for: Dietary Mannan Oligosaccharides Modulate Gut Inflammatory Response and Improve Duodenal Villi Height in Post-Weaning Piglets Improving Feed Efficiency
Source: Animals (Basel). 2020 Jul 28;10(8):1283. doi: 10.3390/ani10081283 (PMC7459834; doi:10.3390/ani10081283)
Supplement: Supplementary file 1 [file animals-10-01283-s001.pdf]

Table S1. Effects of mannan oligosaccharides supplementation in post weaning piglets on GIT villus height to crypt depth ratio and goblet cells. Data shown as LSMeans ± SEM.

Item	Dietary treatment		*p*-Value
	**CON**	**TRT**	
Duodenum			
V:C ratio	0.64 ± 0.02	0.70 ± 0.02	0.15
Goblet cell (nr/villi height)	11.68 ± 1.63	11.68 ± 0.74	0.55
Goblet cell (nr/crypts depth)	30.21 ± 0.96	30.21 ± 1.27	0.20
Jejunum			
V:C ratio	0.76 ± 0.02	0.75 ± 0.02	0.70
Goblet cell (nr/villi height)	6.88 ± 0.97	6.48 ± 0.91	0.55
Goblet cell (nr/crypts depth)	17.94 ± 2.53	18.67 ± 2.66	0.30
Ileum			
V:C ratio	0.83 ± 0.02	0.89 ± 0.02	0.07
Goblet cell (nr/villi height)	13.06 ± 1.84	10.74 ± 1.51	0.07
Goblet cell (nr/crypts depth)	25.45 ± 3.71	23.60 ± 3.40	0.78
Colon			
Goblet cell (nr/crypts depth)	20.69 ± 3.19	22.00 ± 3.27	0.30

Note: CON (n = 10): animals receiving the basal diet with no MOS supplementation; TRT (n = 10): animals receiving the basal diet with 0.2% MOS supplementation; LSMeans: least-square means; SEM: standard error of the mean.
